# Chewing the fat: How lipidomics is changing our understanding of human health and disease in 2022

**DOI:** 10.1002/ansa.202300009

**Published:** 2023-05-10

**Authors:** Caroline Géhin, Stephen J. Fowler, Drupad K. Trivedi

**Affiliations:** ^1^ Manchester Institute of Biotechnology, Department of Chemistry University of Manchester Manchester UK; ^2^ Department of Respiratory Medicine Manchester University Hospitals NHS Foundation Trust Manchester UK; ^3^ School of Biological Sciences, Faculty of Biology, Medicine and Health University of Manchester Manchester UK; ^4^ NIHR Manchester Biomedical Research Centre Manchester University Hospitals NHS Foundation Trust Manchester UK

## Abstract

Lipids are biological molecules that play vital roles in all living organisms. They perform many cellular functions, such as 1) forming cellular and subcellular membranes, 2) storing and using energy, and 3) serving as chemical messengers during intra‐ and inter‐cellular signal transduction. The large‐scale study of the pathways and networks of cellular lipids in biological systems is called “lipidomics” and is one of the fastest‐growing *omics* technologies of the last two decades. With state‐of‐the‐art mass spectrometry instrumentation and sophisticated data handling, clinical studies show how human lipid composition changes in health and disease, thereby making it a valuable medium to collect for clinical applications, such as disease diagnostics, therapeutic decision‐making, and drug development. This review gives a comprehensive overview of current workflows used in clinical research, from sample collection and preparation to data and clinical interpretations. This is followed by an appraisal of applications in 2022 and a perspective on the exciting future of clinical lipidomics.

**Abbreviations**
ACSL4: Acyl‐CoA synthetase long‐chain family member 4
AIDS: Acquired immunodeficiency syndrome
AIF: All‐ion fragmentation
COVID‐19: Coronavirus disease 2019
CVD: Cardiovascular disease
DDA: Data‐dependent acquisition
DI: Direct infusion
DIA: Data‐independent acquisition
ESI: Electrospray ionisation
FA: Fatty acyls
FAB: Fast atom bombardment
FID: Flame ionisation detector
FWHM: Full‐width at half maximum
GC: Gas chromatography
GC‐MS: Gas chromatography‐mass spectrometry
GL: Glycerolipids
GP: Glycerophospholipids
HILIC: Hydrophilic interaction liquid chromatography
HIV: Human immunodeficiency virus
HRMS: High‐resolution mass spectrometry
ICPerMed: International Consortium for Personalised Medicine
(IFN)γ: Interferon gamma
IMS: Ion mobility spectrometry
IRI: Irinotecan
LC: Liquid chromatography
LC‐MS: Liquid chromatography‐mass spectrometry
LIPID MAPS: Lipid Metabolites and Pathways Strategy
LMSD: LIPID MAPS Structure Database
LRMS: Low‐resolution mass spectrometry
LTEC: Long‐term elite controller
MEPS: Micro‐extraction by packed sorbent
MetS: Metabolic syndrome
MRM: Multiple reaction monitoring
MS: Mass spectrometry
NAFLD: Non‐alcoholic fatty liver disease
NMR: Nuclear magnetic resonance
NPLC: Normal‐phase liquid chromatography
OPLS‐DA: Orthogonal projection to latent structure discriminant analysis
PBMC: Peripheral blood mononuclear cell
PC: Lysophosphatidylcholines
PCA: Principal component analysis
PE: Lysophosphatidylethanolamines
PI: Phosphatidylinositols
PK: Polyketides
PKCβII: Protein kinase C βII
PLS‐DA: Partial least squares discriminant analysis
PR: Prenol lipids
PRM: Parallel reaction monitoring
RPLC: Reversed‐phase liquid chromatography
SARS‐CoV‐2: Severe acute respiratory syndrome coronavirus 2
SL: Saccharolipids
SP: Sphingolipids
SPE: Solid‐phase extraction
SRM: Selected reaction monitoring
ST: Sterol lipids
SWATH: Sequential window of all theoretical fragment‐ion spectra
T2D: Type 2 diabetes
TB: Tuberculosis
TSA: Tuberculostearic acid
WHO: World Health Organisation

## 1 INTRODUCTION

Lipids are dynamic, ubiquitous molecules that play vital roles across all biological systems. They perform the following essential functions within cells: energy storage and metabolism, building blocks of cellular and subcellular membranes and intra‐ and inter‐cellular signalling.^1^, ^2^, ^3^, ^4^ Mammalian cells express tens to hundreds of thousands of different lipid species.^5^, ^6^ Endogenous and exogenous factors, such as age, diet, disease, genetics, and environment can influence lipid composition or expression, which means as molecules, they present unique signatures of invaluable information regarding cellular status that is of high interest for the wider scientific community.^4^

The term “lipids” covers a large chemical space of small molecules that are chemically and structurally diverse, varying in aspects such as the length of the hydrocarbon backbone, branching, degrees of unsaturation, and functional groups (Table 1).^7^ These differences occur due to a number of different biochemical transformations during their biosynthesis.^8^ Despite falling under the same umbrella term “*lipids*”, the variability between these molecules is greater than those of genomics and proteomics with an estimated number of structural variations exceeding > 10^5^.^9^, ^10^ Formally, they are defined as “hydrophobic or amphipathic small molecules that may originate entirely or in part by carbanion‐based condensations of thioesters and/or by carbocation‐based condensations of isoprene units”.^11^ Since 2005, the International Lipid Classification and Nomenclature Committee under the sponsorship of the Lipid Metabolites and Pathways Strategy (LIPID MAPS) consortium established the “Comprehensive Classification System for Lipids” that divided all lipids into eight lipid categories: fatty acyls, glycerolipids, glycerophospholipids, sphingolipids, sterol lipids, prenol lipids, saccharolipids, and polyketides (Table 1). As of 13^th^ January 2023, the LIPID MAPS Structure Database (LMSD), which is the largest online lipid database, contains 47,800 unique lipid structures.^12^ Researchers refer to the entire collection of lipid species in a cell, organ, or biological system as a “lipidome” and the large‐scale analytical study of this lipidome as well as the lipids’ interactions with other lipids, proteins, and metabolites as “lipidomics”.^2^, ^3^

**TABLE 1:** Lipid categories as per LIPID MAPS classification system^12^ with example structures. With the exception of various fatty acyls, the logP values taken^13^ show the high hydrophobicity of lipids. The number of molecules in LMSD reported has been updated as of 13^th^ January 2023.

**Lipid category**	**Example structure**	**LogP**	**No. of molecules in LMSD**
Fatty acyls (FA)	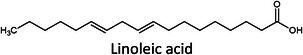	–5–15	10,524
Glycerolipids (GL)	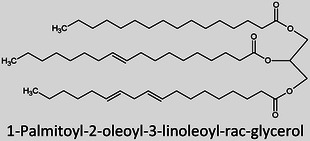	5–35	7,731
Glycerophospholipids (GP)	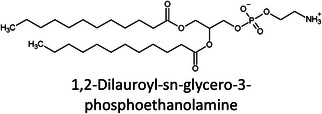	5–25	10,074
Sphingolipids (SP)	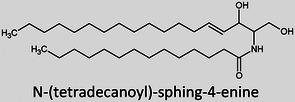	5–25	4,967
Sterol lipids (ST)	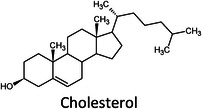	0–20	3,614
Prenol lipids (PR)	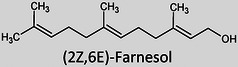	0–20	2,396
Saccharolipids^a^ (SL)	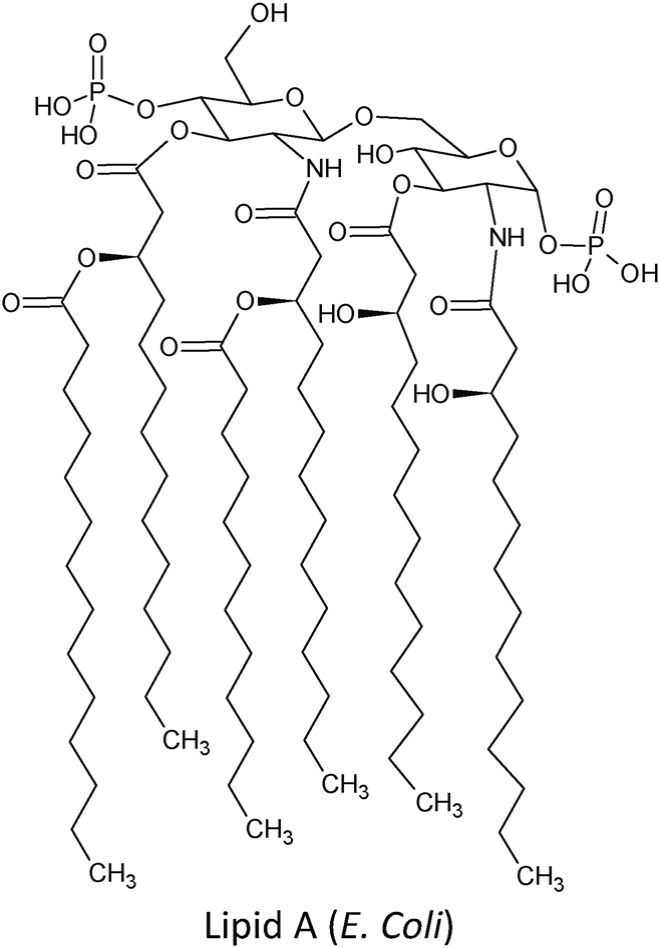	0–30	1,345
Polyketides^a^ (PK)	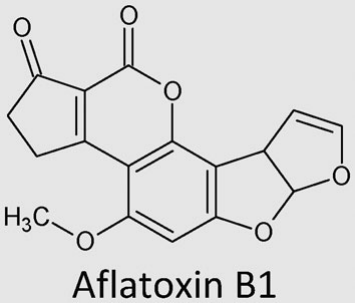	0–15	7,149

^a^ These lipids are not synthesised in mammals and therefore within a clinical context should only represent a small portion of the lipidome.

It was only approximately 40 years ago that lipidomics experiments were made possible due to technological advancements in mass spectrometry (MS). Before this, traditional lipid analysis was performed using techniques such as gas chromatography (GC), high‐performance liquid chromatography, thin‐layer chromatography, nuclear magnetic resonance and other spectroscopic methods, or MS using electron ionisation, chemical ionisation, and field desorption.^14^, ^15^, ^16^, ^17^, ^18^ The major limitations in the past involved addressing the chemical complexity of lipids in biological samples, *i.e.*, the need for instrumentation that can first adequately volatilise and ionise the compounds, and then sensitively analyse and differentiate hundreds of molecules simultaneously. For example, using the molecules shown in Table 1, aflatoxin B1 and 1‐palmitoyl‐2‐oleoyl‐3‐linoleoyl‐rac‐glycerol with boiling points of 528°C^19^ and 801°C,^20^ respectively, are unlikely to readily volatilise in GC without derivatisation, and cholesterol and (2Z,6E)‐farnesol lack ionisable functional groups for efficient soft ionisation. Another notable limitation was the lack of soft ionisation that made intact lipid characterisations and quantification near impossible.

**FIGURE 1:**
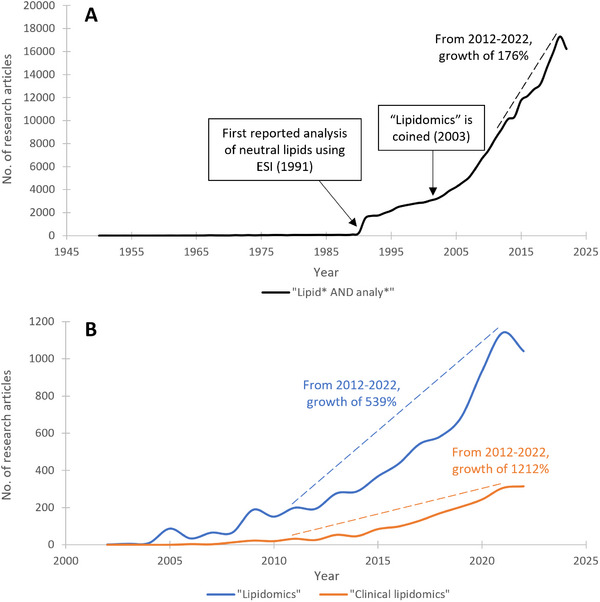
Number of peer‐reviewed research articles published on Web of Science (as of 13^th^ January 2023) using the search terms: (A) “lipid* AND analyte*” between 1950 and 2022, and (B) “lipidomics” and “clinical lipidomics” between 2002 and 2022. Annotated calculations regarding publication growth are calculated as the number of publications in 2022 divided by the number of publications in 2012, multiplied by 100.

The first breakthrough for intact lipid analysis was the development of fast atom bombardment (FAB) by Michael Barber^21^ that showed considerable success for the analysis of polar involatile lipids, such as phospholipids^22^, ^23^ and fatty acids,^24^, ^25^ where lipid class separation and detection with minimal fragmentation was made possible.^22^, ^26^, ^27^, ^28^, ^29^, ^30^ Unfortunately, this was not the case for nonpolar lipids, such as triglycerides, where extensive fragmentation limited its differentiation from other lipid species.^18^ Further, a lack of sensitivity and reproducibility of FAB limited its use in large‐scale analyses. The next breakthrough came through the invention of electrospray ionisation (ESI) by John Fenn in 1989^31^ that greatly accelerated lipid analysis (Figure 1) due to the softer ionisation mechanism, increased ionisation efficiency of nonpolar lipids through adduct formation, and ease of use for quantification.^18^, ^32^, ^33^, ^34^, ^35^, ^36^, ^37^, ^38^, ^39^, ^40^ Now, ESI is the most common ionisation technique used for LC‐MS lipidomics, accounting for nearly two‐thirds of all lipidomics articles since 2010.^41^ Lipidomics is one of today's fastest‐growing research fields (Figure 1)^10^ and it continues to grow through community‐driven advances in technology (MS, chromatography, bioinformatics), creation of shared online lipid databases, and commercial availability of lipid standards.^42^, ^43^

Still in its infancy, the wealth of information offered by lipidomics analysis to understand physiological and pathological processes is currently underutilised in clinics. For the last 60 years, the main manifestation of clinical lipid analyses has consisted of total triglycerides, total cholesterol, low‐density lipoprotein cholesterol, and high‐density lipoprotein cholesterol measurements, which are well established for diagnostics but lack insight concerning the total lipidomic profile.^44^ Clinical lipidomics is an emerging subfield focussed on clinical and consumer access to lipidomics data for disease diagnostics and health management.^44^, ^45^ The main goal of clinical lipidomics is to establish meaningful assays that can inform clinical outcomes. Here, a review of all clinical lipidomics literature published in 2022 found using the Scopus search terms “lipidomics” AND “clinic*” on 15^th^ December 2022 was performed to give a snapshot of the current practices. This review is split into two main parts: 1) a discussion of the overall annual trends identified and how they relate to the conventional lipidomics workflow and 2) a more in‐depth discussion of select research articles on key topic areas.

## 2 2022 IN REVIEW: MATERIALS AND METHODS

### 2.1 Sample collection and experimental considerations

**FIGURE 2:**
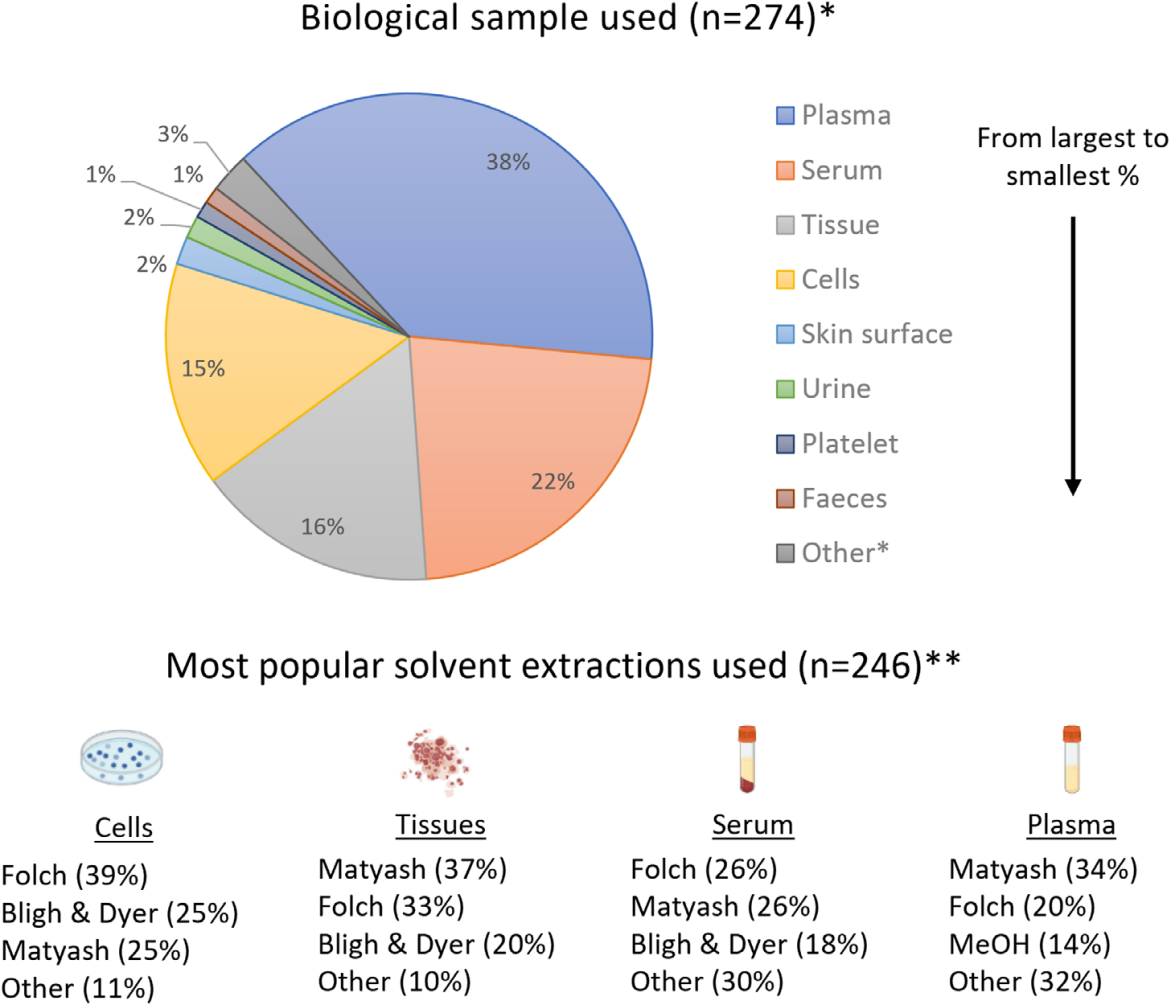
Bibliometric analyses of clinical lipidomics publications in 2022 using Scopus search terms “lipidomics” AND “clinic*” on 15^th^ December 2022.^51^, ^60^, ^61^, ^68^, ^77^, ^155^, ^156^, ^157^, ^166^, ^177^, ^178^, ^179^, ^180^, ^181^, ^182^, ^183^, ^184^, ^185^, ^186^, ^187^, ^188^, ^189^, ^190^, ^191^, ^192^, ^193^, ^194^, ^195^, ^196^, ^197^, ^198^, ^199^, ^200^, ^201^, ^202^, ^203^, ^204^, ^205^, ^206^, ^212^, ^213^, ^214^, ^215^, ^232^, ^233^, ^234^, ^235^, ^236^, ^237^, ^238^, ^239^, ^240^, ^241^, ^242^, ^243^, ^244^, ^245^, ^246^, ^247^, ^248^, ^249^, ^250^, ^251^, ^252^, ^253^, ^254^, ^255^, ^256^, ^257^, ^258^, ^259^, ^260^, ^261^, ^262^, ^263^, ^264^, ^265^, ^266^, ^267^, ^268^, ^269^, ^270^, ^271^, ^272^, ^273^, ^274^, ^275^, ^276^, ^277^, ^278^, ^279^, ^280^, ^281^, ^282^, ^283^, ^284^, ^285^, ^286^, ^287^, ^288^, ^289^, ^290^, ^291^, ^292^, ^293^, ^294^, ^295^, ^296^, ^297^, ^298^, ^299^, ^300^, ^301^, ^302^, ^303^, ^304^, ^305^, ^310^, ^311^, ^312^, ^313^, ^314^, ^322^, ^323^, ^324^, ^325^, ^326^, ^327^, ^328^, ^329^, ^330^, ^331^, ^332^, ^333^, ^334^, ^335^, ^345^, ^346^, ^347^, ^348^, ^349^, ^350^, ^351^, ^352^, ^353^, ^354^, ^355^, ^356^, ^357^, ^358^, ^359^, ^360^, ^361^, ^362^, ^363^, ^364^, ^365^, ^366^, ^367^, ^368^, ^369^, ^370^, ^371^, ^372^, ^373^, ^374^, ^375^, ^376^, ^377^, ^378^, ^379^, ^380^, ^381^, ^382^, ^383^, ^384^, ^385^, ^386^, ^387^, ^388^, ^389^, ^390^, ^391^, ^392^, ^393^, ^394^, ^395^, ^396^, ^397^, ^398^, ^399^, ^400^, ^401^, ^402^, ^403^, ^404^, ^405^, ^406^, ^407^, ^408^, ^409^, ^410^, ^411^, ^412^, ^413^, ^414^, ^415^, ^416^, ^417^, ^418^, ^419^, ^420^, ^421^, ^422^, ^423^, ^424^, ^425^, ^426^, ^427^, ^428^, ^429^, ^430^, ^431^, ^432^, ^433^, ^434^, ^435^, ^436^, ^437^, ^438^, ^439^, ^440^, ^441^, ^442^, ^443^, ^444^, ^445^, ^446^, ^447^, ^448^, ^449^, ^450^, ^451^, ^452^, ^453^, ^454^, ^455^, ^456^, ^457^. *Note: All biological sample types with more than 1.0% of total publications have been reported individually with all remainder combined under “Other”. **Note: For trending purposes, the unmodified and modified versions of the classic methods (Folch, Bligh & Dyer and Matyash) were combined. The sample images have been created using BioRender.com.

A wide variety of biological samples can be used for clinical lipidomics analysis. The samples chosen are often driven by the clinical question being investigated. Biofluids are commonly studied for biomarker discovery, whereas tissues or cells are commonly used to investigate physiological mechanisms.^46^ In 2022, as shown in Figure 2, the most popular samples were plasma, serum, and tissue, accounting for 38%, 22%, and 16% of all publications, respectively. Blood samples contain rich signatures due to their sensitivity to the effects of disease, environment, nutrition, and genetic variation. They are also routinely obtained from patients and stored in biobanks,^47^ making them readily available for scientific investigations.

The method and timing of sample collection can have a significant impact on the downstream lipid profile. Fasting status and circadian rhythms for blood samples, for example, have been shown to influence lipidomes.^48^, ^49^, ^50^ To minimise the intra‐ and inter‐variation of cohorts, most studies sample blood at the fasting state to measure baseline levels; however, profiling in the postprandial state could also prove useful. For instance, Velenosi et al. analysed pre‐ and postprandial human plasma samples using standardised mixed meals of control and non‐alcoholic fatty liver disease (NAFLD) patients by untargeted lipidomics. Here, insulin‐related increases in multiple diacylglycerol species at 4 h postprandially were found specifically in NAFLD patients and not in controls, which have been linked to the hepatic secretion of endogenous and not meal‐derived lipids.^51^

Sample handling and storage will also cause variations in lipid profiles. For blood samples, the choice of anticoagulants and collection tubes has been demonstrated to have an impact on lipid extraction and MS ionisation.^52^, ^53^, ^54^, ^55^, ^56^, ^57^, ^58^, ^59^ Two studies performed by Beger et al. and Yadav et al. in 2022 investigated the effects of formalin fixations on the untargeted lipid profiles of brain and skin tissue samples, respectively.^60^, ^61^ Both studies highlight how preservation added bias to the lipid classes observed, resulting in reduced quality data; therefore, any preservation used should be considered by scientists prior to their study regardless of sample type. After sampling, tissues should be immediately frozen in liquid nitrogen and any biofluids should either be processed immediately or stored at −80°C to limit enzymatic activity or chemical degradation.^41^ The multitude of factors that can impact the sampled lipidomes stresses the importance of optimal sampling and storage.

Appropriate experimental design is crucial for clinical lipidomics due to the large intra‐ and inter‐individual biological variability of the human lipidome, resulting in further disease heterogeneity.^62^, ^63^, ^64^ Small sample sizes or the lack of suitable study participants can lead to underpowered designs, resulting in inconclusive or misleading results. Power calculations for discovery experiments are necessary, but difficult with unknown effect sizes, to account for technical and biological variance.^65^, ^66^ This is especially true for small‐cohort studies where the variability could obscure trends or lead to high false discovery rates. Bias and confounding factors (e.g. differences in storage conditions, testing sites, sampling time) should be factored into the experimental design. Uncontrollable factors (e.g. ethnicity and diet) should also be accounted for in the study design through replication, randomization, and blocking schemes.^67^ Further consideration of the clinical application of your study should influence your study design to answer questions such as: what patient population would benefit from these biomarkers, or in which clinical settings will these tests be used?^68^ This would aid the translation of research to clinics, where aspects such as costs, facilities available, transportation, and patient demographics may be of relevance.^45^

### 2.2 Sample preparation

After the sample has been collected, it typically undergoes pre‐treatment before analysis for multiple reasons: 1) extraction from biological material, 2) sample matrix clean‐up, 3) analyte preconcentration, and 4) compatibility with instrumentation. For untargeted analyses, where profiling the entire lipidome is of interest, it is important to consider the sampling bias introduced by the extraction method chosen.^69^, ^70^, ^71^, ^72^, ^73^, ^74^, ^75^, ^76^, ^77^ This is a bottleneck in lipidomics workflows due to the large structural diversities of lipids present in biological matrices. The most suitable extraction method for the highest coverage should be investigated through research or literature review prior to experimentation. The amount of sample could also influence the extraction selected.^69^, ^78^ For targeted approaches, sample preparation is a key area to optimise to make substantial gains in method performance. Here, pre‐treatment approaches, such as solid‐phase extraction (SPE) or derivatisation, for example, esterification, silylation, or charge‐switch derivatisation, can drastically increase analyte sensitivity and/or decrease matrix effects.^41^, ^79^, ^80^, ^81^, ^82^, ^83^ Disadvantages associated with both SPE and derivatisation include their costs, time‐consuming, and cumbersome nature, as well as issues with repeatability due to either batch‐to‐batch variation in sorbents or reagents and/or analyst consistency.^78^, ^84^, ^85^, ^86^, ^87^, ^88^

Solvent extractions are the gold standard in lipidomics sample preparation. Historically, the most widely used solvent extraction systems are the biphasic chloroform/methanol/water mixtures, e.g. “Folch”^89^ and “Bligh & Dyer”^90^ protocols; however, these classical methods are becoming superseded by new liquid extraction methods, such as the biphasic “Matyash”^91^ or “BUME” protocols, or alternatively monophasic alcoholic solutions using isopropanol or methanol, due to their reduced toxicity, automation potential, costs, and/or ease of use.^2^, ^69^, ^76^, ^91^, ^92^, ^93^, ^94^, ^95^, ^96^, ^97^ Due to the large sample sizes used, automation of sample preparation is highly advantageous for speed, throughput, and reproducibility.^92^, ^96^, ^97^ With or without the automation of the sample preparation, it is crucial that solvent selection is not only driven by chemistry but also by eco‐friendliness. Analytical studies should aim to develop selective extraction methods to promote green chemistry.^98^, ^99^, ^100^ This shift has been reflected in the trends observed for 2022, where the Folch and Matyash protocols competed to be the most popular extractions for cells, tissues, serum, and plasma (Figure 2). Interestingly, a new monophasic extraction protocol has been developed in 2022 by Fu et al. for untargeted human platelet extraction utilising methanol/methyl tert‐butyl ether/isopropanol (1.3:1:1 *v/v/v*), reporting almost 100% extraction recoveries of polar and nonpolar lipids. With large‐scale clinical lipidomics studies in mind, the advantages of their solvent system include practicality, eco‐friendliness (reduced solvent consumption and no halogenated solvents), speed, and adaptability to other biofluids.^77^

Lastly, the use of internal standards is key to monitoring extraction efficiency (e.g. recovery and repeatability) and accounting for any analyte losses downstream. In 2022, ∼27% of research publications used internal standards.^1^ Ideally, there should be an internal standard representative of the analyte being quantified, such as an isotopically labelled lipid standard; however, this is not practically possible for untargeted methods involving thousands of compounds. Here, the standardisation of analyte responses is typically performed through the use of commercially available, synthetic lipid standards from AVANTI Polar Lipids through the LIPID MAPS initiative to aid lipid quantification and identification.^42^, ^43^ The final extract is typically evaporated and reconstituted prior to the introduction to the analytical platform.

### 2.3 Data acquisition

#### 2.3.1 Separation sciences

**FIGURE 3:**
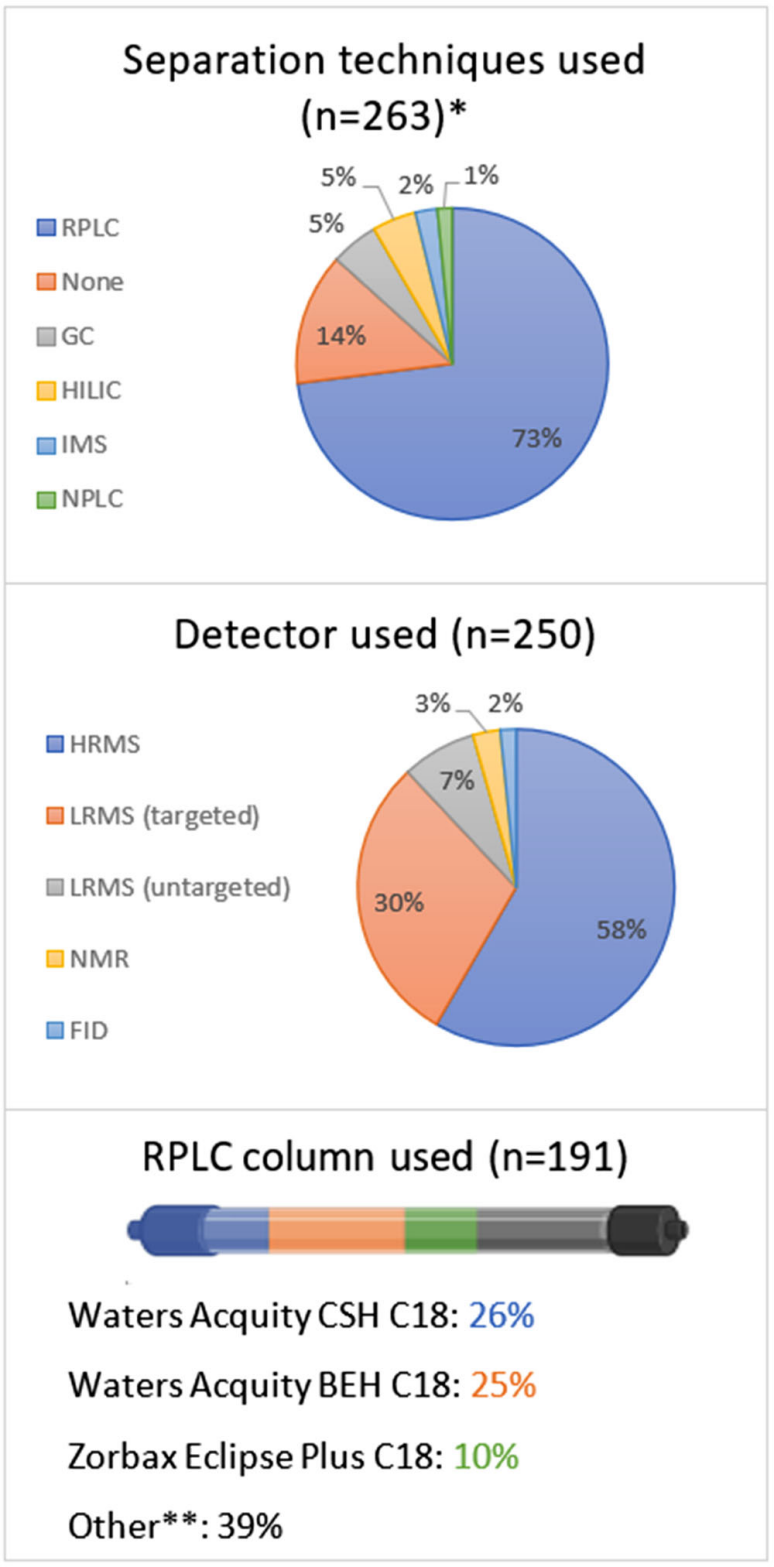
Bibliometric analyses of clinical lipidomics publications in 2022 according to separation techniques, detectors, and RPLC columns used.^51^, ^60^, ^61^, ^68^, ^77^, ^155^, ^156^, ^157^, ^166^, ^177^, ^178^, ^179^, ^180^, ^181^, ^182^, ^183^, ^184^, ^185^, ^186^, ^187^, ^188^, ^189^, ^190^, ^191^, ^192^, ^193^, ^194^, ^195^, ^196^, ^197^, ^198^, ^199^, ^200^, ^201^, ^202^, ^203^, ^204^, ^205^, ^206^, ^212^, ^213^, ^214^, ^215^, ^232^, ^233^, ^234^, ^235^, ^236^, ^237^, ^238^, ^239^, ^240^, ^241^, ^242^, ^243^, ^244^, ^245^, ^246^, ^247^, ^248^, ^249^, ^250^, ^251^, ^252^, ^253^, ^254^, ^255^, ^256^, ^257^, ^258^, ^259^, ^260^, ^261^, ^262^, ^263^, ^264^, ^265^, ^266^, ^267^, ^268^, ^269^, ^270^, ^271^, ^272^, ^273^, ^274^, ^275^, ^276^, ^277^, ^278^, ^279^, ^280^, ^281^, ^282^, ^283^, ^284^, ^285^, ^286^, ^287^, ^288^, ^289^, ^290^, ^291^, ^292^, ^293^, ^294^, ^295^, ^296^, ^297^, ^298^, ^299^, ^300^, ^301^, ^302^, ^303^, ^304^, ^305^, ^310^, ^311^, ^312^, ^313^, ^314^, ^322^, ^323^, ^324^, ^325^, ^326^, ^327^, ^328^, ^329^, ^330^, ^331^, ^332^, ^333^, ^334^, ^335^, ^345^, ^346^, ^347^, ^348^, ^349^, ^350^, ^351^, ^352^, ^353^, ^354^, ^355^, ^356^, ^357^, ^358^, ^359^, ^360^, ^361^, ^362^, ^363^, ^364^, ^365^, ^366^, ^367^, ^368^, ^369^, ^370^, ^371^, ^372^, ^373^, ^374^, ^375^, ^376^, ^377^, ^378^, ^379^, ^380^, ^381^, ^382^, ^383^, ^384^, ^385^, ^386^, ^387^, ^388^, ^389^, ^390^, ^391^, ^392^, ^393^, ^394^, ^395^, ^396^, ^397^, ^398^, ^399^, ^400^, ^401^, ^402^, ^403^, ^404^, ^405^, ^406^, ^407^, ^408^, ^409^, ^410^, ^411^, ^412^, ^413^, ^414^, ^415^, ^416^, ^417^, ^418^, ^419^, ^420^, ^421^, ^422^, ^423^, ^424^, ^425^, ^426^, ^427^, ^428^, ^429^, ^430^, ^431^, ^432^, ^433^, ^434^, ^435^, ^436^, ^437^, ^438^, ^439^, ^440^, ^441^, ^442^, ^443^, ^444^, ^445^, ^446^, ^447^, ^448^, ^449^, ^450^, ^451^, ^452^, ^453^, ^454^, ^455^, ^456^, ^457^ All legends are in order of largest to smallest percentage. *Note: All techniques that represent ≥1.0% of total publications have been reported. **Note: all "Other" named columns constituted ≤5% of publications. The column image has been created in BioRender.com. Key: NPLC = normal‐phase liquid chromatography, RPLC = reversed‐phase liquid chromatography, HILIC = hydrophilic interaction liquid chromatography, GC = gas chromatography, IMS = ion mobility spectrometry, HRMS = high‐resolution mass spectrometry, LRMS = low‐resolution mass spectrometry, FID = flame ionisation detector, NMR = nuclear magnetic resonance.

The complexity of biological matrices can be reduced through the implementation of separation prior to detection. For lipidomics, this is usually performed using either GC or liquid chromatography (LC) (Figure 3). These techniques can potentially separate isomers and isobars, reduce matrix effects, increase low abundance analyte sensitivities, and minimise the reliance on the detector for quantification and identification purposes. This, however, comes at the expense of lower sample throughput and complicated data processing in comparison to direct infusion approaches.^2^ Unconventional approaches include two‐dimensional chromatography^101^, ^102^, ^103^, ^104^, ^105^ or ion mobility spectrometry.^106^, ^107^, ^108^, ^109^

GC separates compounds based primarily on differences in volatility combined with interactions with the stationary phase.^110^ Lipids generally have low volatility and for any carboxylic acids, there can be interactions with stationary phase siloxane groups that result in undesirable late‐eluting broad peaks. To combat this, derivatisation approaches, such as transesterification to fatty acid methyl esters, silylation to trimethylsilyl esters and charge‐switch derivatisation, are performed.^41^, ^79^, ^80^, ^81^, ^82^ Derivatisations are laborious and tend to complicate interpretation due to the presence of derivatisation artifacts^111^; however, a key advantage of derivatisation is its selectivity. Derivatisation typically targets specific chemical functionalities, which means that for complex biological samples, this theoretically could limit the number of analytes in GC‐MS chromatograms, allowing easier interpretation and quantification.

**FIGURE 4:**
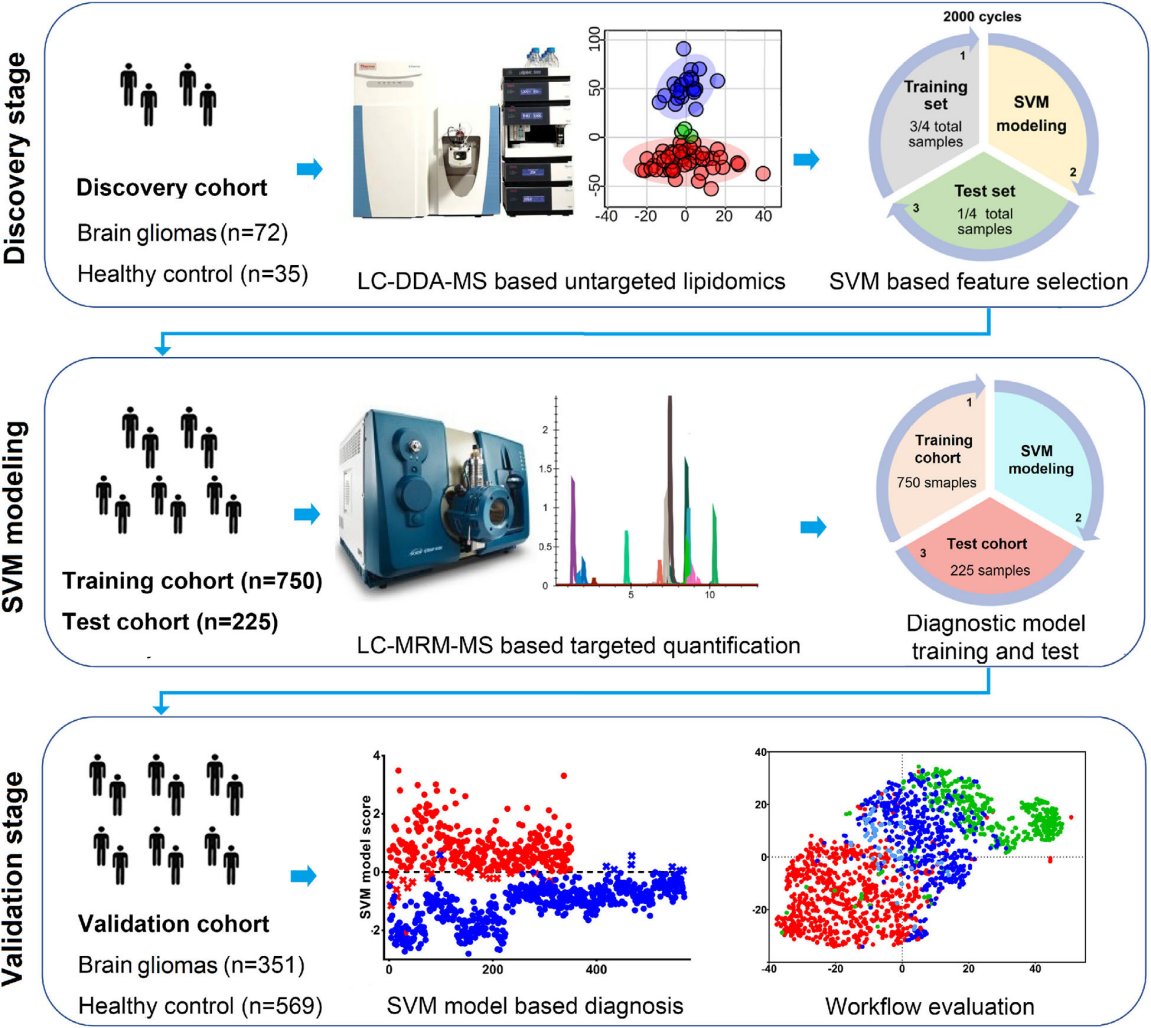
The study design and development of a liquid chromatography‐mass spectrometry (LC‐MS) method from initial biomarker discovery to validation for the diagnosis of malignant brain gliomas. Towards the left of every panel, notice the increase in cohort size as the method enter validation to make it more meaningful for clinical implementation. In the middle of the first two panels, see how the discovery was performed using untargeted high‐resolution MS (HRMS) analysis for screening all lipids and showing clear discrimination between healthy versus disease through a principal component analysis (PCA) plot, whereas the clinical method uses a simpler, targeted method for quantification. Finally, towards the right of the first two panels, see how machine learning through support vector machine modelling is applied, where the model is trained and validated after each study. Reprinted with permission.^155^

Unlike GC, LC is not limited to volatile compounds. LC separates compounds based on interactions of the sample with a mobile and stationary phase. There are many different types of LC, but generally, reversed‐phase LC (RPLC) for nonpolar separations and hydrophilic interaction LC (HILIC) for polar separations are the most popular. As a rule of thumb, RPLC will retain analytes with a logP ≥ ‐1 and HILIC will retain analytes with a logP ≤ 1.^112^, ^113^ Based on the physicochemical properties of lipids (Table 1), it comes as no surprise that RPLC continues to be the most popular separation technique, accounting for 73% of the literature published in 2022 (Figure 4).

RPLC uses a nonpolar stationary phase and a polar aqueous mobile phase to generally elute analytes in order of decreasing polarity. Analytes are separated according to two phenomena, namely partitioning and/or adsorption via hydrophobic interactions to the bonded stationary phase. The degree to which each phenomenon contributes to an analyte's retention varies but has been shown through molecular simulations to be dependent on stationary phase alkyl chain length and functional group type.^114^ For lipidomics, the retention order predominantly relies on the analyte alkyl chain length and degree of unsaturation.^115^ The most popular RPLC columns in 2022 were the Acquity CSH column (26%), the Acquity BEH C18 column (25%), and the Zorbax Eclipse Plus C18 (10%) (Figure 3).

HILIC uses a polar stationary phase and a nonpolar organic‐rich mobile phase to generally elute analytes in order of increasing polarity. It is a variant of normal‐phase LC (NPLC) and complementary to RPLC. Due to a water‐enriched layer adsorbed onto the stationary phase, HILIC separates ions using a mixed‐mode retention mechanism. This mechanism will involve, to different degrees, the following phenomena: hydrophilic partitioning between the mobile phase and the water layer, adsorption to the stationary phase, and/or electrostatic interactions and hydrogen bonding with the stationary phase moieties. The retention order predominantly relies on the lipid headgroup polarity.^106^, ^116^ Example lipids analysed by HILIC include ceramides, glycerolipids, glycolipids, phospholipids, and sphingolipids.^102^, ^106^, ^116^, ^117^, ^118^, ^119^

#### 2.3.2 Detection

Due to its high sensitivity and specificity, it comes as no surprise that MS was the most popular detector in 2022, accounting for an overall 95% of the literature published in 2022 with high‐resolution MS (HRMS) making up 58% and low‐resolution MS (LRMS) making up 37% (Figure 3). MS is an analytical technique that creates gas‐phase ions from atoms or molecules and measures their mass‐to‐charge ratios (*m/z*).^120^ Mass spectrometers discriminate between ions of different *m/z* by subjecting them to constant, pulsed, or periodically time‐varying electric and/or magnetic fields.^121^ For lipidomics, mass spectrometers are the detectors of choice, where they are capable of (but not limited to): 1) molecular mass, elemental, and isotopic composition determinations, 2) structural elucidation, and 3) quantification. HRMS is commonly defined as mass spectrometers that are capable of resolving powers ≥ 10,000 full‐width at half maximum (FWHM). Generally, HRMS, such as time‐of‐flight and orbitraps, would be used for untargeted analyses due to their higher resolving powers that allow accurate mass measurements, improvements in signal:background ratios, and wider mass ranges. For targeted applications and quantitative approaches, LRMS, such as triple quadrupoles in tandem, are commonly used for their high specificity, sensitivity, and wide linear dynamic ranges.

There are three main mass spectrometric approaches: direct‐infusion MS (termed “shotgun” lipidomics), chromatographically coupled MS (typically LC or GC), or MS imaging (MSI). Key aspects that could influence the approach chosen include matrix effects, confidence in identification, and ease of quantification. Matrix effects, in particular, ion suppression by more dominant and/or easily ionisable lipid species, such as phospholipids,^122^, ^123^, ^124^, ^125^ is a major challenge in lipidomics. This problem is more pronounced for shotgun lipidomics as all lipid species are analysed simultaneously, which consequently leads to detection problems concerning low‐level lipid species. Ion suppression can be overcome by either incorporating chromatography or derivatisation.^41^, ^79^ It is important to note that LC (RPLC or HILIC) can readily separate different lipid classes and therefore resolve their lipid‐lipid interactions and suppression (i.e., interclass interactions), but within‐class interactions (i.e., intraclass interactions) could be significantly increased and this could counterproductively result in greater ion suppression at large concentrations.^101^, ^126^ In terms of identifications, shotgun lipidomics and MSI offer less confidence due to the lack of molecular separation and retention time information; whereas, through chromatographic coupling, ions are separated from one another prior to detection adding confidence in identification at the expense of more chemical waste and low throughput due to longer analytical run times. Finally, for quantification, in shotgun lipidomics, the steady ionisation environment allows robust quantification using a lipid standard mix with one compound of each lipid class. This approach is dissimilar to chromatographically coupled MS or MSI because the ionisation environment is constantly changing. This means that more thorough calibration work is needed for quantification.^80^ Further, MSI analyses lipids in situ with the key advantage of capturing spatial information about the analyte.^127^

For untargeted screening methods, the HRMS acquisition modes typically used are full scan, data‐dependent acquisition (DDA), and data‐independent acquisition (DIA). Full scan is an MS^1^ approach that obtains all *m/z* measurements across a defined *m/z* window. In DDA mode, the HRMS performs a full scan on MS^1^ followed by an MS^2^ analysis of select precursor ions for structural information. This selection is intensity‐based, which means that the DDA data are biased and lower‐intensity lipids will therefore not have any associated MS^2^ data. In DIA mode, the HRMS performs a full scan on MS^1^ followed by an MS^2^ analysis of all precursor ions through instrument cycles. Commonly used DIA methods include all‐ion fragmentation (AIF) or sequential window of all theoretical fragment‐ion spectra (SWATH).^128^ The main advantage of DIA is that it is less biased than DDA and there is no undersampling of peaks. Therefore, all precursor peaks are fragmented; however, this results in more complicated MS/MS spectra, often losing the connection between the product ions to their precursor due to ion overlap.^129^, ^130^ Several studies highlight the loss of 2‐5x sensitivity when using DIA versus DDA or full scan modes.^131^, ^132^, ^133^ For more information, the following reviews are recommended.^129^, ^131^, ^134^

Based on information from discovery experiments and/or literature, targeted lipidomics experiments can be performed, where a pre‐defined set of lipids with a known *m/z* of precursor and product ions are analysed in a quantitative manner across large sample sets. Here, selected or multiple reaction monitoring (SRM/MRM) on triple quadrupole instrumentation, and recently parallel reaction monitoring (PRM) on quadrupole time‐of‐flight and orbitraps are employed.^135^ Specialised tools, such as LipidCreator^136^ and METLIN‐MRM,^137^ can aid experimental method development and processing steps. In comparison to untargeted data processing (discussed below), the post‐acquisition processing of targeted data is relatively straightforward using open‐access tools, such as Skyline^138^ or XCMS‐MRM,^137^ or vendor software, such as TargetLynx,^139^ that can automate peak integration, relative and absolute quantification, and evaluate data quality.^140^

### 2.4 Data processing

The high‐throughput nature of lipidomics and the ‘big data’ generated, in particular for untargeted experiments, results in high complexity analysis that has become heavily reliant on statistical testing, mathematical treatment, and computational algorithms.^141^, ^142^ First, raw data are typically converted to a data format that is compatible with downstream processing through either commercial software packages, such as Progenesis QI,^143^ or freely available ones, such as MSConvert that allow a direct conversion of raw data that can be read by software programs, such as MetaboAnalyst,^144^ after deconvolution using software such as MZmine,^145^ or packages such as XCMS^146^ or eRah^147^ in R. These programs use algorithms for spectral filtering, baseline correction, peak alignments, normalisation, peak picking, and putative annotations. An example of these programs is the peak detection tool “NeatMS”, which was published by Gloaguen et al. in 2022, to combat the current challenges of irreproducibility and peak overpicking experienced in *omics* data post‐acquisition. It is an open‐source automated tool that uses machine learning to better differentiate between chemical signals and noise that can readily be added to existing workflows.^148^

Then, advanced biostatistical tools are applied to analyse and interpret the data in the context of clinical information. After all, the assumption behind the collection of these lipidomics data is that the co‐variation of a small set of analyte abundances should inform the biological phenomenon being studied. The challenge here is to identify and isolate the lipids that are specific to the phenomenon from the ones that are not relevant. Supervised or unsupervised statistical methods are used. Briefly, these include (but are not limited to) approaches, such as partial least squares discriminant analysis (PLS‐DA), orthogonal projection to latent structure discriminant analysis (OPLS‐DA), and principal component analysis (PCA).^142^, ^149^ From this, analytes of interest observed to be specific to the biological phenomenon can be further identified through automated programmatic approaches to allow database searching^140^ and/or subjected to pathway analysis. Through pathway analyses, the dysregulated lipids observed can be associated with specific metabolic pathways, potentially representing the body's response to a disease or drug treatment (Figure 6).^150^, ^151^

Finally, as part of the big data revolution, machine learning has gained popularity for building models, e.g. diagnostic tests, to help translate research into clinical practice. Machine learning is the study of computational algorithms that make predictions and decisions based on experience and data, without being explicitly programmed.^152^ It is an approach to data analysis that involves building and adapting models to “learn” from experience to further improve their ability to make predictions.^153^, ^154^ A good example of machine learning applications as applied to biomarker discovery is shown in the articles by Zhou et al. (Figure 4)^155^ and Wang et al.^156^ Machine learning algorithms, such as lasso (or ridge) regressions, decision trees, support vector machines (ensembles or wrappers of these), and artificial neural networks, are powerful tools for pattern recognition or classification that have significantly accelerated the identification of biomarkers. Interestingly, in Luan et al.’s biomarker discovery work studying spontaneous miscarriage in IVF‐ET women, they approach machine learning in a systematic approach, whereby five classifiers and four feature selection methods were optimised to give the best classification model with the highest accuracy and stability.^157^

## 3 2022 IN REVIEW: RESULTS AND DISCUSSION

It is known that many diseases are characterised by lipid dysregulation. Lipidomics articles within clinical research generally have two main objectives: to understand molecular mechanisms of lipid metabolisms or establish diagnostic biomarkers. Biomarker studies aim to translate to routine clinical practice; whereas, physiological mechanism studies lend themselves to understanding disease manifestations, identifying therapeutic drug targets, and aiding drug development. To translate this to clinical lipidomics, these profiles need to be integrated with patient phenotypes to potentially identify disease severity‐, duration‐, stage‐, subtype‐ and prognosis‐specific lipidome changes.^45^

**FIGURE 5:**
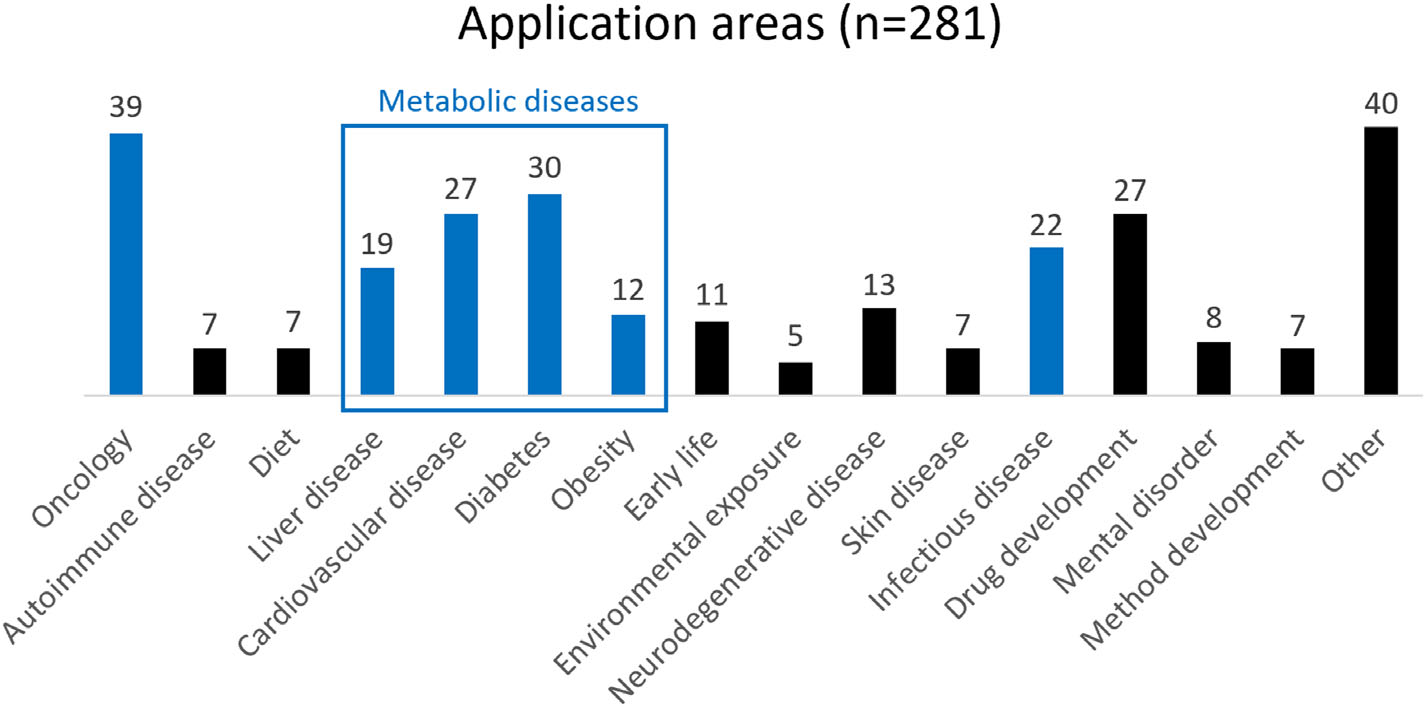
Application areas with ≥ 5 publications have been represented. Application areas with < 5 publications have been sorted under “Other”. *Note: “Early life” combines pregnancy and infant lipidomics studies. Application areas in blue are discussed in this review.

A biomarker is defined as: “*A defined characteristic that is measured as an indicator of normal biological processes, pathogenic processes, or biological responses to an exposure or intervention, including therapeutic interventions*”.^158^ An ideal biomarker should be: 1) binary or at least quantifiable, 2) the testing should have a turnaround speed appropriate to the clinical indication and be adoptable into routine clinical practice, 3) highly sensitive and/or specific and 4) readily detectable in a non‐invasive biofluid. There are essential method development steps (Figure 4) to establish biomarkers for a disease, namely biomarker discovery and validation. A panel of biomarkers may be used for better performance in terms of diagnostic accuracy.^159^, ^160^, ^161^, ^162^, ^163^ In the following sections, selected research articles from the most popular subject areas of 2022 will be briefly discussed: oncology, metabolic diseases (e.g. cardiovascular disease (CVD), diabetes, liver disease, and obesity), and infectious diseases (Figure 5).

### 3.1 Oncology

Cancer is the second largest cause of death worldwide with an overall incidence rate of 20.2% over a human lifetime.^164^ It has been shown that lipid metabolism in cells is drastically elevated during the different stages of cancer development.^165^, ^166^, ^167^ Increased lipids are needed for plasma membrane synthesis and energy production, but also influence cell membrane compositions to favour metastasis and trigger signalling events within cancer cells. Further, these increased lipids also facilitate metabolic crosstalk between the tumour and surrounding cells within the tumour microenvironment.^165^, ^167^ The upregulation of fatty acids and cholesterol are common in cancer as their synthetic pathways help facilitate cell proliferation^168^ and tumour growth,^169^ respectively. Further, lysophospholipid levels have been associated with cancer cell migration, invasion ability of cancer cells, and potentially tumour immunity.^170^, ^171^, ^172^, ^173^ Lipidomic analyses have revealed cancer‐specific lipid dysregulations that could potentially serve as promising biomarkers for screening purposes,^174^, ^175^ as demonstrated by Lee et al. in a plasma analysis of 140 human samples that could differentiate five cancers (liver, lung, gastric, colorectal, and thyroid).^176^ It is therefore of no surprise with its known lipid dysregulation and urgency that oncology was the biggest application area in 2022, where the following cancers were studied: bladder,^177^ brain,^155^ breast,^178^, ^179^, ^180^, ^181^, ^182^ colorectal,^183^, ^184^, ^185^, ^186^, ^187^, ^188^ endometrial,^189^ gallbladder,^190^ gastric,^191^ kidney,^192^ liver,^193^, ^194^, ^195^, ^196^, ^197^ lungs,^156^, ^198^, ^199^, ^200^ nasopharyngeal,^201^ ovarian,^202^ paediatric,^203^ prostate,^204^, ^205^ and skin.^206^

To maximize the survival rates of cancer patients, it is important to identify cancer at its early stages, where tumours can be surgically removed or treated with milder drug regimens.^207^ The average 5‐year survival rate in the early stages is 91%; whereas, the average 5‐year survival rate in the late stages is 26%.^208^ The most common cancers for men are prostate (20%), lung and bronchus (13%), and colorectal (9%) cancers; whereas, for women, they are breast (30%), lung and bronchus (13%), and colorectal (8%) cancers.^208^ The “harder to suspect” cancers due to non‐specific symptoms that typically require multiple consultations that delay diagnosis are multiple myeloma, pancreatic cancer, ovarian, stomach, and lung cancers.^209^ Wang et al. discovered for early‐stage lung cancer that the lipid metabolism was dysregulated with the glycerophospholipid metabolism being the most altered. For biomarker discovery, untargeted RPLC‐HRMS analysis of plasma samples revealed nine key lipids for early‐stage cancer detection. Using these, a targeted RPLC‐MS/MS method with a run time of 19 min was developed to separate and quantify these lipids. This assay reached 100% specificity on the validation cohort, and subsequently, out of 1,145 participants from different medical centres, achieved more than 90% sensitivity and 92% specificity.^156^

Tumour formation is characterised by the successful circumvention of cell death regulation to allow unlimited replication and immortality.^210^ Ferroptosis is a newly identified iron‐dependent form of cell death driven by lipid peroxidation that has garnered interest in the scientific community for its potential as an alternative cancer therapy.^211^ Traditional tumour treatments, such as chemotherapy and radiotherapy, are often not as effective due to the breadth of their targets and tumour cell tolerance.^210^, ^211^ Ferroptosis activation may be a potential strategy for treating cancers that are unsuccessfully targeted by conventional therapies. Zhang et al. used untargeted RPLC‐HRMS analyses of cells to study the molecular mechanisms of ferroptosis, namely the sensors and their link to the amplification process of lipid peroxidation. The enzyme protein kinase C βII (PKCβII) was found critical in ferroptosis in its role of phosphorylation and activating the enzyme Acyl‐CoA synthetase long‐chain family member 4 (ACSL4) required for the lipid peroxidation. The activation of the PKCβII‐ACSL4 pathway could be a potential therapeutic target.^212^ Further, Liao et al. showed that T cell‐derived interferon (IFN)γ with arachidonic acid‐induced tumour ferroptosis through the activation of ACSL4 pathway by several methods, including a targeted phospholipid analysis using NPLC‐MS/MS. These data demonstrated how T cells can selectively reprogram tumour cells via the IFNγ signalling pathway. Potential supplementation of arachidonic acid could be an effective therapy for tumour regression.^213^

To understand how lipidomics could be used to influence treatment decisions, three studies focusing on drug development will be discussed. Chen et al. demonstrated using untargeted ambient single‐cell HRMS that metformin, an oral antidiabetic drug, has a synergistic effect with the cancer drug irinotecan (IRI) to overcome the drug resistance of previously IRI‐resistant colorectal cancer cells. This was done by studying the metabolome changes in model systems of drug‐resistant cancer cells with both metformin monotherapy and metformin/IRI co‐therapy. Metformin monotherapy induced downregulation of lipids and fatty acids, potentially through the inhibition of fatty acid synthase, whereas co‐treatment resulted in reduced production of ceramide phosphoethanolamine.^214^ Antona et al. explored drug repurposing to identify new potential therapies for colorectal cancer that are overall quicker and cheaper than de novo drug synthesis. Spiperone, an antipsychotic drug, was shown to have anticancer effects by inducing apoptosis in colorectal cancer cells. The drug action involved phospholipase C activation, intracellular calcium homeostasis dysregulation, and endoplasmic reticulum stress induction, which caused significant lipid metabolism alterations. This study used several techniques including untargeted RPLC‐HRMS to study drug‐induced changes to the overall cancer cell lipidome.^186^ Hatfield et al. analysed the pretransplant serum of patients receiving allogeneic stem cell transplantation to treat haematological malignancies using untargeted DI‐MS/MS. These procedures have a high risk of treatment‐related morbidity and mortality. Through hierarchical clustering analyses, associations between pretransplant lipid profiles and posttransplant complications (including death due to disease relapse or treatment toxicity) were found, demonstrating that patients could be subclassified prior to transplants to direct therapies and inform treatment outcomes according to the individual.^215^

### 3.2 Metabolic diseases

Metabolic diseases occur when abnormal chemical reactions disrupt the body's metabolism. Due to modern sedentary lifestyles and changed nutritional behaviours, they are becoming increasingly prevalent worldwide and currently pose a large burden on global healthcare systems.^216^, ^217^ The major metabolic diseases include type 2 diabetes (T2D), NAFLD and metabolic syndrome (MetS). Of these, T2D affects more than 200 million people^216^ and NAFLD affects an estimated one‐third of the adult population worldwide.^218^ There has been an alarming increase in MetS^219^, ^220^ with an estimated prevalence in 1/4^th^ of the global population.^221^, ^222^ MetS, also known as syndrome X,^223^ insulin resistance syndrome,^224^ and the deadly quartet,^225^ refers to the co‐occurrence of several cardiovascular risk factors, such as glucose intolerance, obesity, insulin resistance, hypertension and dyslipidaemia.^226^ MetS is closely associated with the development of T2D, fatty liver, CVD, and cancer.^227^, ^228^, ^229^, ^230^, ^231^ Due to the elevated risk of mortality with these diseases, there is a need for strategies in its identification, prevention, and treatment within healthcare. In 2022, as evidenced in Figure 5, the bulk of research articles focussed on related diseases: CVD,^232^, ^233^, ^234^, ^235^, ^236^, ^237^, ^238^, ^239^, ^240^, ^241^, ^242^, ^243^, ^244^, ^245^, ^246^, ^247^, ^248^, ^249^, ^250^, ^251^, ^252^, ^253^, ^254^, ^255^ diabetes,^166^, ^233^, ^249^, ^250^, ^256^, ^257^, ^258^, ^259^, ^260^, ^261^, ^262^, ^263^, ^264^, ^265^, ^266^, ^267^, ^268^, ^269^, ^270^, ^271^, ^272^, ^273^, ^274^, ^275^, ^276^, ^277^, ^278^ liver disease,^51^, ^279^, ^280^, ^281^, ^282^, ^283^, ^284^, ^285^, ^286^, ^287^, ^288^, ^289^, ^290^, ^291^, ^292^, ^293^ MetS^277^, ^294^, ^295^ and obesity.^277^, ^295^, ^296^, ^297^, ^298^, ^299^, ^300^, ^301^, ^302^, ^303^, ^304^, ^305^

**FIGURE 6:**
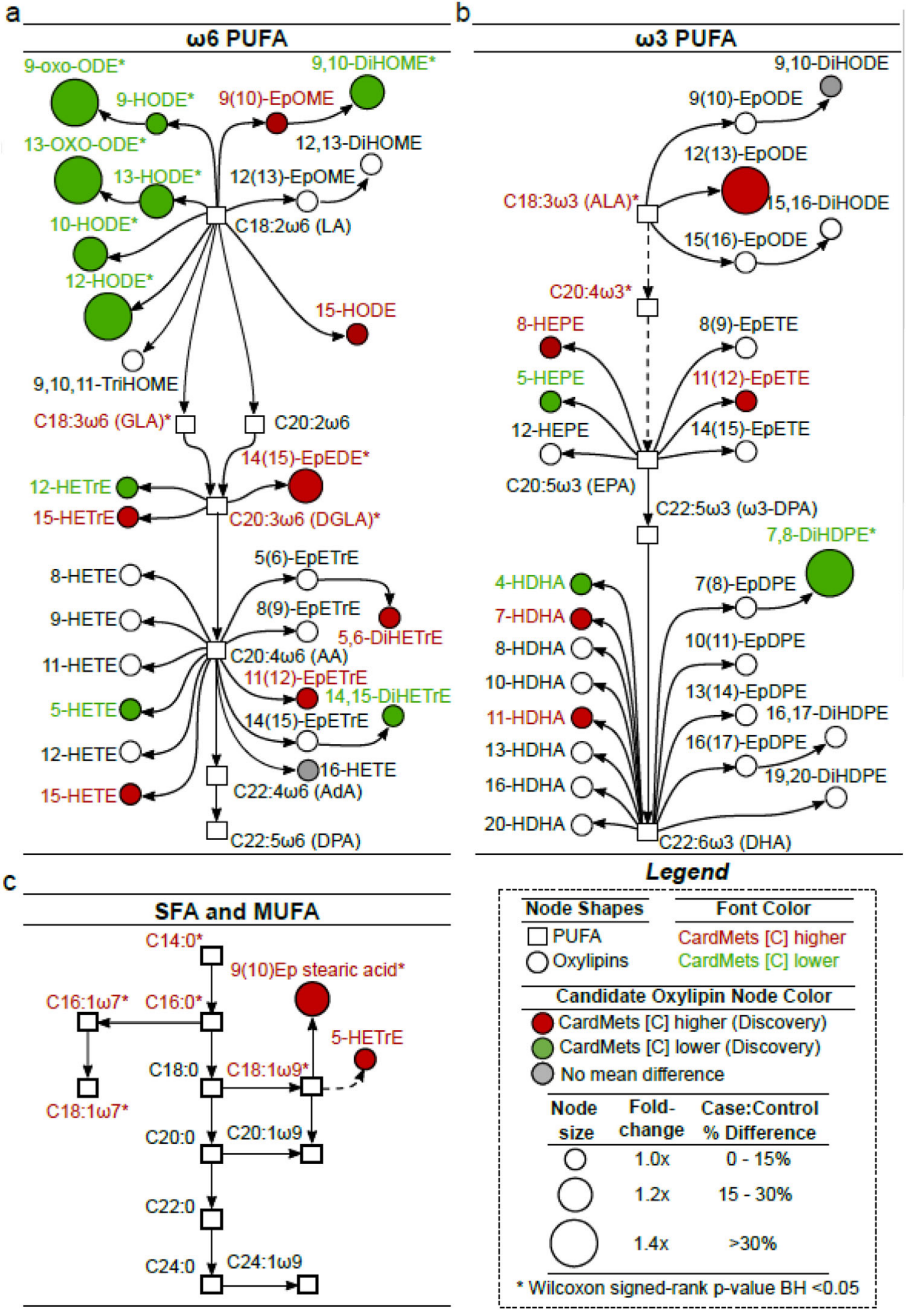
Pathway analysis of oxylipins (*n* = 54; circles) and fatty acids (*n* = 25; squares) identified that can discriminate patients with MetS from controls. The metabolic pathways of oxylipin biosynthesis from omega‐6 PUFA (A), omega‐3 PUFA (B), and SFA/MUFAs (C). The oxylipins of interest from the analytical testing are highlighted in colour, where red = upregulated in metabolic syndrome patients, green = downregulated in metabolic syndrome patients, and grey = no difference. Dashed lines represent indirect pathways including intermediate metabolites. Size of nodes represents fold changes between the two cohorts. Reprinted with permission.^294^

Coleman et al. investigated the pathogenesis and involvement of the gastrointestinal tract with the syndrome through multiple experiments including an untargeted RPLC‐MS/MS analysis of faecal samples of people with MetS and healthy controls. No signs of intestinal inflammation or increased permeability were found in the MetS patients compared to the controls; however, significant increases in 417 lipid features, including various glycerolipids, glycerophospholipids, sphingolipids, fatty acyls, and polyketides, in the gut lipidome were observed, suggesting alterations of the intestinal host‐microbiota metabolism.^277^ Further, Dalle et al. quantitatively analysed plasma for over 130 oxylipins of 476 participants using two orthogonal analytical platforms for added confidence: targeted RPLC‐MS/MS and GC‐MS using fatty acid methyl ester derivatisation. This work demonstrates how a comprehensive understanding of oxylipins as lipid mediators, regulating many cardiometabolic functions, can help elucidate MetS pathophysiology through effective pathway analysis (Figure 6). Some of these oxylipins are directly linked to key molecular pathways, including oxidative stress, inflammation, blood clotting, glucose homeostasis, and adipogenesis, providing additional information that is not provided by the current clinical diagnostic tests. A classification model of 23 oxylipins was developed to stratify patients with MetS at risk of cardiometabolic diseases.^294^

Several groups performed large‐cohort longitudinal lipidomics experiments to develop models for disease prognosis. By studying the plasma samples of 4,067 participants over 23 years using DI‐HRMS, Lauber et al. applied machine learning models to develop a lipidomics‐based risk score using 184 lipid concentrations to assess a patient's future risk of developing T2D, CVD, and obesity. Individual lipidomes are stratified into different classifications (low‐high risk) such that interventions could be introduced years before disease incidence for prevention.^233^ Al‐Sari et al. used a combination of clinical measurements and omics analyses of plasma samples of 537 participants with type 1 diabetes over 5 years to develop risk scores using machine learning approaches to predict a patient's progression to diabetes complications, such as diabetic kidney disease or diabetic retinopathy. Lipidomic analyses were performed using untargeted RPLC‐HRMS. Thus, the models, using a combination of clinical measurements, metabolites, and lipids, have the potential for the early detection and prevention of complications.^276^

### 3.3 Infectious diseases

Infectious diseases are illnesses caused by microorganisms, such as bacteria, viruses, fungi, or parasites, that are transmitted from an infected person, animal, or object to a susceptible host. According to the World Health Organisation (WHO), they cause an estimated quarter of all deaths worldwide^306^ and this number is only expected to grow with climate change, issues with global food supply chains, and international travel that allow these diseases to spread. Globally, it is the leading cause of childhood mortality.^307^ As lipids combine with other metabolites and specific pathways to support the human lifecycle, these compounds are involved in the interplay between the infectious agent and its host. This includes how pathogens become internalised into the host's cells, interactions between the pathogen and the host's lipid metabolism and microbiota, and the immune system.^308^, ^309^ Malaria, tuberculosis, and Human immunodeficiency virus/acquired immunodeficiency syndrome (HIV/AIDS) remain within the 10 top causes of death in low‐ to middle‐income countries.^306^ These diseases have been subject to clinical lipidomics experiments to further disease understanding and manifestation.^310^, ^311^, ^312^, ^313^, ^314^

Tuberculosis (TB) is a highly contagious airborne disease, dating back more than 9,000 years,^315^ caused by the bacteria *Mycobacterium tuberculosis*. In 2020, the WHO reported an estimated 10 million new tuberculosis cases and 1.5 million TB‐related deaths globally with two‐thirds of the cases spread across eight countries: India, China, Indonesia, Philippines, Pakistan, Nigeria, Bangladesh, and South Africa.^316^ Brandenburg et al. showed by using a targeted shotgun HRMS analysis that tuberculostearic acid (FA 19:0; TSA)‐containing phosphatidylinositols (PI) are molecular markers of infection with clinically relevant *M. tuberculosis* complex strains and signify bacterial burden. They developed an indirect, culture‐free targeted lipid assay for bacterial cultures that can be performed within a day (30x faster than conventional methods) to measure the pathogen loads of *M. tuberculosis* in infected murine microphages, human neutrophils, and murine lung tissue. These PIs were found to be enriched in peripheral blood mononuclear cells (PBMCs) from active TB patients. The focus on PBMC analysis instead of blood analysis was chosen to avoid variation caused by the metabolic state of patients.^310^

HIV is a virus that attacks the body's immune system with the most advanced infection stage being AIDS.^317^ According to the WHO, there were 1.5 million new HIV cases and approximately 650,000 HIV‐related deaths in 2021.^318^ Sub‐Saharan Africa contains a disproportionate amount of HIV cases, accounting for more than 70% of all global cases.^318^, ^319^, ^320^ Masip et al. studied plasma samples using untargeted RPLC‐HRMS of long‐term elite controllers (LTECs), a small subset of HIV individuals that have long‐term viral and immunological HIV control that are used as models of a functional cure, using untargeted RPLC‐HRMS. Here, a panel of 45 lipids could differentiate the clinical outcome of LTEC individuals on whether they are losing viral and/or immunological control, which could inform therapeutic interventions.^311^ Wang et al. analysed plasma samples using RPLC‐HRMS of women with or at high risk of HIV to study alterations in host gut microbiota upon infection. The data together with network analysis showed positive associations between enriched pathogenic gut bacteria *Fusobacterium* and *Proteus* to the host lipidomic and metabolomic profiles, particularly in lysophosphatidylcholines (PC), lysophosphatidylethanolamines (PE), and diglycerides, which are associated with the progression of atherosclerosis.^252^

Severe acute respiratory syndrome coronavirus 2 (SARS‐CoV‐2) is a positive‐sense RNA virus responsible for the global coronavirus disease 2019 (COVID‐19) pandemic that started in 2019 with more than 660 million people infected and more than 6.7 million deaths (on 12^th^ January 2023).^321^ Since its outbreak in 2019, the world population has experienced a drastic change in lifestyle due to the unpredictable nature of the disease. Despite the explosive efforts by the scientific community, there remain many questions regarding its pathological mechanisms, treatment, and the clinical management of patients that research groups have attempted to address through lipidomics experiments.^322^, ^323^, ^324^, ^325^, ^326^, ^327^, ^328^, ^329^, ^330^, ^331^, ^332^, ^333^, ^334^, ^335^

Castañé et al. analysed serum using RPLC‐HRMS in a semi‐quantitative approach to compare the lipidomic signatures of hospitalised COVID‐19 patients against disease controls (other infectious/inflammatory diseases, e.g. CVD, diabetes, kidney diseases and cancer) and healthy controls. Clear differences in acylcarnitines, fatty acids, and oxylipins were observed between COVID‐19 and healthy patients, but more importantly, this study shows how many of these lipid alterations were not specific to COVID‐19 patients and was shared with other infectious/inflammatory diseases. Altered levels in oxylipins, bile acids, and glycerophospholipids were found to best distinguish between COVID‐19 and negative controls.^323^

Biagini et al. quantified the plasma levels of 48 oxylipins and five cytokines using micro‐extraction by packed sorbent (MEPS)‐RPLC‐MS/MS of hospitalised COVID‐19 ward and ICU patients to study their inflammatory responses to COVID‐19, in particular the cytokine and oxylipin storms.^336^ Here, ICU patients were found to have significantly lower levels of oxylipins, suggesting an impaired inflammatory response that would put them at higher risk of severe viral attacks.^328^ Similarly, Karu et al. quantified the plasma levels of 63 signalling lipids (free fatty acids, oxylipins and their intermediates, and immune‐active endocannabinoids) by RPLC‐MS/MS to understand COVID‐19 severity and progression. Substantial differences between the signalling lipid profiles of COVID‐19 patients were found at varying disease stages. The metabolic picture of severe patients was correlated with persistent inflammation and a disruption in the balance of signalling lipids, which could potentially prevent an effective shift into the resolution of inflammation.^330^

Finally, Saud et al. studied the molecular composition of the SARS‐CoV‐2 lipid envelope using virus particles by HILIC‐MS/MS. This knowledge is important to design anti‐viral agents as well as improve our understanding of viral‐host interactions, infectivity, pathogenicity, and immune system clearance. The SARS‐CoV‐2 lipid membrane was observed to be primarily composed of PC, PE, and PI with a high proportion of external aminophospholipids. The molecular fatty acyl species may vary depending on the host cell. Further, in vitro and randomised controlled clinical studies of COVID‐19 patients using surfactant‐containing mouthwashes were found to be a potential antiviral approach for wider prevention and societal control strategies.^322^

### 3.4 Future perspectives

In this review, we have demonstrated the relevance of lipidomics data for the elucidation of potential biomarkers and disease mechanisms in clinical studies. With future development, these could improve the diagnosis and prognosis of various diseases and be used to predict and measure treatment efficacy. They are largely not yet ready to be used in a clinical setting, as reflected by the low number of *omics*‐based biomarkers that have been approved by regulatory agencies and used in clinical settings,^337^, ^338^ and consequently, *omics* technologies are often perceived as over‐promising and/or underdelivering in clinical applications. The main challenges here include the inappropriate design of clinical trials and lack of validation,^339^, ^340^ and the difficulty and lack of standardisation of analytical methods.^339^, ^340^, ^341^ With the number of lipidomics research articles concerning biomarker discovery continually increasing, there is a growing demand to convert these complex multivariate signatures into reliable cost‐effective assays that can routinely be used in practice. Lipidomics plays a key role here in first discovering a biomarker in a small cohort, then trialling its suitability through larger, validation cohorts with diverse participants for representation and inclusion, before translating its detection to a cheaper reliable method that can be used by the wider community at a clinic or at home (exemplified in Figure 4).^342^

An exciting opportunity for clinical lipidomics is precision medicine (also known as personalised medicine), which aims to provide individualised healthcare in terms of disease treatment and prevention. It involves assessing the genotype and/or phenotype of a patient before treatment to more accurately and comprehensively direct therapy,^340^, ^343^ acknowledging the importance of an individual's characteristics in response to treatment. It would rework the current healthcare practices, from the one‐size‐fits‐all approach typically after a patient reports symptoms to one that tests a patient with an ability to preventatively predict diseases and determine which medical treatments will be safest and most effective for the individual patient.^343^ This approach is more likely to detect non‐communicable diseases. According to Leroy Hood, a pioneer in this area, precision medicine promises to 1) provide deep insights into disease mechanisms, 2) make blood a diagnostic window for both the health and disease of an individual, 3) stratify complex diseases into subtypes, 4) provide new ways to drug target discovery, and 5) generate metrics for evaluating wellness.^341^ It aims to be preventative, predictive, personalised, and participatory (termed “P4 medicine”).^341^ According to the International Consortium for Personalised Medicine (ICPerMed), the implementation of precision medicine is targeted to be before 2030.^344^

## 4 CONCLUSION

In conclusion, the most popular clinical research areas of 2022 in lipidomics were oncology, metabolic diseases (e.g. CVD, diabetes, liver disease and obesity), drug development, and infectious diseases. This is likely due to both their urgency and their known lipid dysregulations. MS‐based lipid analyses and sophisticated bioinformatics approaches are growing in parallel and can revolutionise lipidomic research. Still in its infancy, lipidomics has a strong potential to change the face of current clinical practices for disease stratification and precision medicine approaches; however, clear hurdles, such as the difficulty to convert complex laboratory procedures to simple reliable tests, lack of clinical sizes and validation cohorts for regulatory approval, prevent its current translation.

## AUTHOR CONTRIBUTIONS

Caroline Géhin: Conceptualisation, investigation, visualisation, and writing—original draft. Stephen J. Fowler: Reviewing and editing. Drupad K. Trivedi: Conceptualisation, reviewing, and editing.

## CONFLICT OF INTEREST STATEMENT

The authors declare no conflict of interest.

## Data Availability

Data sharing is not applicable to this article as no new data were created or analysed in this study.

## Footnotes

1 The bibliometric analysis was performed on the Google Scholar search engine (10^th^ April 2023) using two searches: 1) an in‐text search of the keywords “lipidomics clinic*” limited to research articles in 2022 and 2) an in‐text search of the keywords “lipidomics clinic*” AND “internal standard*” limited to research articles in 2022. A percentage was calculated by dividing the number of publications for search #2 by search #1, then multiplying by 100.
